# The Brain’s Microvascular Response to High Glycemia and to the Inhibition of Soluble Epoxide Hydrolase Is Sexually Dimorphic

**DOI:** 10.3390/nu14173451

**Published:** 2022-08-23

**Authors:** Saivageethi Nuthikattu, Dragan Milenkovic, Jennifer E. Norman, John Rutledge, Amparo Villablanca

**Affiliations:** 1 Division of Cardiovascular Medicine, University of California, Davis, CA 95616, USA; 2 Department of Nutrition, University of California, Davis, CA 95616, USA

**Keywords:** multi-omics, microvascular, brain, dementia, sex difference, high glycemic diet, soluble epoxide hydrolase inhibitor, males, females

## Abstract

Biological sex and a high glycemic diet (HGD) contribute to dementia, yet little is known about the operative molecular mechanisms. Our goal was to understand the differences between males and females in the multi-genomic response of the hippocampal microvasculature to the HGD, and whether there was vasculoprotection via the inhibition of soluble epoxide hydrolase (sEHI). Adult wild type mice fed high or low glycemic diets for 12 weeks, with or without an sEHI inhibitor (t-AUCB), had hippocampal microvessels isolated by laser-capture microdissection. Differential gene expression was determined by microarray and integrated multi-omic bioinformatic analyses. The HGD induced opposite effects in males and females: the HGD-upregulated genes were involved in neurodegeneration or neuroinflammation in males, whereas in females they downregulated the same pathways, favoring neuroprotection. In males, the HGD was associated with a greater number of clinical diseases than in females, the sEHI downregulated genes involved in neurodegenerative diseases to a greater extent with the HGD and compared to females. In females, the sEHI downregulated genes involved in endothelial cell functions to a greater extent with the LGD and compared to males. Our work has potentially important implications for sex-specific therapeutic targets for vascular dementia and cardiovascular diseases in males and females.

## 1. Introduction

Brain vascular dysfunction is one of the components of neurological and chronic metabolic diseases, such as Alzheimer’s disease (AD), vascular dementia (VaD), stroke, and diabetes mellitus (DM) [1,2,3,4,5]. Among the 5.8 million Americans with AD, two thirds are female [6]. Females have a later onset but more rapid cognitive decline after a diagnosis of dementia than males [7]. In contrast to AD, males are at an increased risk of developing VaD than females [8], although the reasons are not known. As per the Alzheimer’s Disease Research Network, there is increasing evidence that biologic sex is associated with disease risk as well as the development and progression of dementia [9,10]. However, the role sex plays in the etiology and prognosis of the disease is still unclear. Hence, it is important to focus on sex in order to better understand the molecular mechanisms of disease that will aid in developing therapies perhaps more specific to men and women.

Cardiovascular disease (CVD) and AD vascular pathology affect primarily the macro- and microvascular circulations, respectively [11,12]. DM greatly increases the risk of CVD and cerebrovascular diseases [5]. In addition, females are at greater risk of developing vascular disease from DM than males [13,14,15]. Since it is increasingly recognized that CVD and AD share common risk factors, including DM, hypertension, and hyperlipidemia [16,17,18], our work has focused on understanding the molecular changes in gene expression patterns in the microvessels of brain memory centers in both males and females, and characterizing the molecular sex differences in these CVD risk factors. The comparison of the differentially expressed genes, pathways, and transcriptional and post-transcriptional regulatory mechanisms in males and females is crucial for identifying potential sex-specific drugs and therapeutic targets for the leading causes of death. Nevertheless, sex comparisons alone are not sufficient, and there is a need for the additional step of a comprehensive sex/gender-based analysis [19,20,21].

Therefore, it is essential to sex-disaggregate data since we do not understand the different and unique sex differences in the vascular determinants of dementia and how a key risk factor, like hyperlipidemia or hyperglycemia, exerts control over the brain microvascular genome in both the sexes. Utilizing animal models, we previously identified and defined the sex-dependent molecular mechanisms of lipotoxic injury in brain microvasculature in the LDL-R -/- (low density lipoprotein receptor knock out) murine model utilizing the Western diet [9,22,23]. In these studies, we showed that following lipid injury, male mice had a much more limited molecular genetic response to lipid stress, whereas female mice were able to engage a greater number and variety of more neuroprotective cellular pathways, including the regulation of angiogenesis and endothelial cell proliferation and migration. We also showed that females had less severe cognitive dysfunction compared to males under hyperlipidemic stress. The female-specific differential expression of transcription factors were associated with VEGF signaling, angiogenesis, and protection from stress-related apoptosis. In contrast, the differential expression of male specific transcription factors resulted in cellular signaling involved in promoting vascular injury, cell death, neurodegeneration, and neuro-inflammation.

However, the Western diet contains both elevated fat and glycemic content making it difficult to discern the individual contribution of these dietary components, which is necessary to better understand their specific effects. Unfortunately, there is a dearth of literature on high glycemic diets (HGD) in the absence of high fat content. Recently, we demonstrated for the first time that the HGD induces a detrimental transcriptomic response in the microvessels of the hippocampus in males [24] and in females (submitted manuscript in revision). In males, these transcriptomic changes proceed via upregulated pathway-associated transcription factors, protein coding, and noncoding genes involved in microvascular dysfunction, oxidation, inflammation, and alterations in mitochondrial function. In contrast, in females on the HGD, the majority of the differentially expressed genes were downregulated and involved in cellular pathways that promote deleterious cellular functions, such as hyperpermeability, apoptosis, and neurovascular inflammation, and impaired endothelial cell barrier, migration, and angiogenesis. We have also previously reported that treatment with a soluble epoxide hydrolase inhibitor (sEHI) reversed many of the transcriptomic changes induced by a HGD in the hippocampal microvasculature of male mice [24]. sEH is an enzyme responsible for conversion of the neuroprotective and vasodilatory epoxyeicosatrienoic acids (EETs) into less biologically active dihydroxyeicosatrienoic acids (DHETs) [25,26]. Surprisingly, the sEH inhibitor modified the transcriptome of female mice consuming a low glycemic diet (LGD) over an HGD by modulating genes involved in metabolic pathways that synthesize neuroprotective EETs and associated with a higher EETs/DHETs ratio.

Hence, the aim of this study was to perform a comparative sex analysis of the global multi-genomic changes induced by a high-glycemic diet and a sEH inhibitor in the brain microvasculature of hippocampus of male and female mice and identify potential sex-specific molecular mechanisms. Defining this for the entire mouse genome in males and in females under comparable and replicable study conditions is absolutely key to doing more precise and relevant health related research that attends to sex as a fundamental biologic variable with relevance to dementia, cardiovascular diseases, and diabetes. Furthermore, comparing the differentially expressed genes, pathways, and transcriptional and post-transcriptional regulatory mechanisms identified in our previous studies in males and females is crucial for identifying potential sex-specific drug targets for hyperglycemia-induced microvascular endothelial injury relevant to vascular dementia. Thus, our current work is a comparative analysis of the sex-specific mechanisms that may render vasculo-protection from hyperglycemic stress in females compared to males.

## 2. Materials and Methods

### 2.1. Experimental Animals, Diet, and Soluble Epoxide Hydrolase Inhibitor (sEHI) Treatment

Male mice (1 per cage) and female mice (2 to 3 per cage) were housed in the University of California, Davis Mouse Biology Program, throughout this study with a 12-h light/dark cycle in a temperature- and humidity-controlled environment. Throughout the study, food and water were provided ad libitum except when indicated as part of experimental procedures. Vivarium staff monitored food intake, water, and activity. The study was carried out in compliance with the policy of Public Health Service on the Humane Use and Care of Laboratory Animals and the University of California, Davis Institutional Animal Care and Use Committee (IACUC), approved protocol number 20943 on 18 April 2019.

C57BL/6J male and female mice (Jackson Laboratories, stock 000664) began the study at 19 weeks of age and were given a standard chow diet (catalog no 0915 from Envigo Teklad Diets, Madison, WI, USA) and acclimated for one week before starting the study procedures. In order to provide a chronic model of hyperglycemic stress, 20-week-old Wildtype mice were fed a high glycemic diet (HGD) or a control low glycemic diet (LGD) for 12 weeks. The composition of the experimental diets are detailed in Supplemental Table S1. For male and female mice, we had four experimental treatment groups (*n* = 7 mice per group): LGD only, LGD with soluble epoxide hydrolase inhibitor (LGD + sEHI), HGD only, and HGD with sEHI (HGD + sEHI). Mice received a soluble epoxide hydrolase inhibitor (sEHI), t-AUCB (trans-4-[4-(3-adamantan-1-yl-ureido)-cyclohexyloxy]-benzoic acid, Cayman Chemical, Ann Arbor, MI, USA) at a concentration of 10 mg/L in the drinking water using 1% (by volume) polyethylene glycol 400 (PEG400) (Millipore, Burlington, MA, USA) [27]. Mice consumed approximately 7 to 7.5 mL of water each day, consistent with previously published work [28] and a daily dose of 2.5 to 3 mg of t-AUCB (sEHI) per kg. At the end of the 12-week dietary intervention, mice were sacrificed at 32 weeks of age. Body weight was measured at baseline and at the completion of the experimental diet exposure.

### 2.2. Serum Glucose, and Insulin Assays

For the pre-diet samples, blood was drawn via submandibular nick. For the post-diet samples, blood was drawn at the time of sacrifice through ventricular puncture. For both samples, mice were fasted for eight hours prior to blood collection, blood was stored at −80 °C. Glucose, and insulin concentrations were measured in the fasted serum samples. Glucose concentration was measured through enzymatic assays from Fisher Diagnostics (Middleton VA, USA), and insulin concentrations were measured via electrochemiluminescence from Meso Scale Discovery (Rockville, MD, USA) in accordance with the manufacturer’s protocols. The UC Davis Mouse Metabolic Phenotyping Center (MMPC) Metabolic Core conducted all serum assays in this study.

### 2.3. Vaginal Lavage and Assessment of Estrus Cycle

At the end of the 12-week dietary intervention period, male and female mice were anesthetized by intraperitoneal xylazine/ketamine. On female mice, vaginal lavage was performed using sterile phosphate buffered saline (PBS) prior to sacrifice. The PBS with vaginal cells was then streaked on a glass slide and stored at room temperature. The phase of the estrus cycle was assessed by the staining of the cells collected by the lavage with 0.1% crystal violet and examining the stained cells under a light microscope. Based on the cell types identified, samples were categorized as proestrus, estrus, metestrus, or diestrus, as described by McLean et al. [29].

### 2.4. Isolation and Cryosection of Murine Brain Hippocampus

Tissue was collected at the end of the 12-week dietary intervention during sacrifice via euthanasia by exsanguination under anesthesia. Intact brains were quickly isolated and sliced into regions comprising the temporal lobe segment containing the hippocampus and embedded in HistoPrep Frozen Tissue Embedding Media (Fisher Scientific, Pittsburgh, PA, USA) under RNAse free conditions. The hematoxylin staining of the brain sections in the medial temporal lobe allowed the identification of the hippocampus and hippocampal neurons and visualization via microscopy as formerly described [30]. Coronal cryosection was then performed on the hippocampus (8 µm, Leica Frigocut 2800n Cryostat, Leica Biosystems, Buffalo Grove, IL, USA), and samples were captured on charged RNA-free PEN Membrane Glass slides, covered with RNAlater^®^-ICE (Life Technologies, Grand Island, NY, USA) to preserve RNA, and kept at −80 °C until needed.

### 2.5. Laser Capture Microdissection (LCM) of Hippocampal Microvessels

Hippocampal cryosections were coated in nuclease-free water and dehydrated in desiccant when ready for LCM. Samples were stained using Alkaline phosphatase with 5-bromo-4-chloro-3-indolyl phosphate/nitro blue tetrazolium chloride (BCIP/NBT) substrate to mark hippocampal endothelial microvessels (<20 um) in the cryosections, which would later be subject to transcriptomic analysis as previously described [31]. Then, laser capture microdissection (LCM) allowed for the extraction of the microvascular endothelium from hippocampal cryosections. Specifically, the capture of the entire vessel wall was performed under direct microscopic visualization using a Leica LMD6000 Laser Microdissection Microscope (Leica Microsystems, Wetzlar, Germany). While microvessels largely represent endothelial enriched regions in hippocampus dorsal segments that would have contained CA1 and CA3 regions, they were not specified by hippocampal region or subregion.

### 2.6. RNA Extraction from Laser Captured Brain Microvessels

The laser-captured hippocampal microvessels (300 microvessels per mice, *n* = 3 mice per experimental group) were subjected to total RNA extraction utilizing an Arcturus PicoPure™ RNA Isolation Kit (Thermo Fisher Scientific, Santa Clara, CA, USA) in accordance with the manufacturer’s instructions. RNA quantification was performed as per Affymetrix RNA quantification kit with SYBR Green I and ROX™ Passive Reference Dye protocol from Affymetrix, Santa Clara, CA, USA, and Nanodrop was used to determine RNA quality of LCM-derived microvessels. After the RNA preparation of all the tissue for microarray analysis, a total of 122.3 picograms of RNA was used per array per mouse.

### 2.7. Microarray Hybridization and Transcriptome Analysis

Clariom D Mouse Array was utilized, which includes more than 7 million probes for protein and non-protein coding genes, including microRNAs (miRNAs), long non-coding RNAs (LncRNAs), and small nucleolar RNAs (snoRNAs) from Thermo Fisher, Santa Clara, CA, for transcriptomics analysis. Three arrays were used for each diet/inhibitor group. An amount of 122.3 pico grams of RNA (122.3 pg) was used to generate cRNA and sscDNA with GeneChip^®^ WT Pico Kit (Thermo Fisher, Santa Clara, CA, USA). The total amount of RNA extracted from 300 brain hippocampal microvessels of each animal was sufficient to obtain the required amount of sscDNA (5.5 ug) suggested by the manufacturer’s protocol for microarray analysis. We fragmented the SscDNA using uracil-DNA glycosylase (UDG) and apurinic/apyrimidinic endonuclease 1 (APE 1) and labeled it with terminal deoxynucleotidyl transferase (TdT) using the biotin-linked DNA Labeling Reagent. The hybridization, staining, and scanning of the fragmented and labeled sscDNA samples was conducted by the UC Davis Genome Center shared resource core, according to the Thermo Fisher Scientific WT array hybridization protocol. The hybridization of the fragmented and labeled sscDNA samples was performed with the GeneChip™ Hybridization Oven 645, and then the GeneChip™ Fluidics Station 450 was used to wash and stain the samples. The arrays were scanned with GeneChip™ Scanner 3000 7G (Thermo Fisher Scientific, Santa Clara, CA, USA). Thermo Fisher Scientific Transcriptome Analysis Console software version 4.0.2 quality-checked the data analysis and microarrays. We have deposited the female and male mice microarray data in GEO, and the accession numbers are GSE195975 and GSE185057, respectively.

### 2.8. Bioinformatic Analysis

Sex- and Gender-Based Analysis (SGBA) is an approach that systematically examines the sex-based (biological) and gender-based (socio-cultural) determinants of health and disease. For these studies, SGBA was conducted by incorporating sex (male/female) as a biologic variable into our research design, disaggregating data by sex in the collection, analysis, and interpretation of data, as well as by looking for intersectional relationships [32].

To assess the sex differences in the brain hippocampal microvasculature, we first carried out bioinformatic analyses of differentially expressed genes (DEGs) in order to compare male versus female response to hyperglycemic stress. We also studied the sex differences in the multi-omic response of the sEH inhibitor in the presence of the low or high glycemic diets. We compared the following study groups: HGD vs. LGD, LGD + sEHI vs. LGD, and HGD + sEHI vs. HGD in female and male mice. Two of the study investigators (SN and DM) utilized multiple software to perform the bioinformatic analysis of DEGs from these three diet/inhibitor comparison groups identified using Thermo Fisher Scientific (Santa Clara, CA, USA) Transcriptome Analysis Console software version 4.0.2.

Metaboanalyst [33,34] was used to generate the principal component analysis (PCA) plot of the identified differentially expressed genes (DEGs) and the variable importance in projection (VIP) scores were obtained from partial least squares-discriminant analysis (PLS-DA). ClustVis [35] was utilized to perform hierarchical clustering. To identify the different types of RNAs, ShinyGo online database [36] was used. Enrichr [37] helped to identify transcription factors and disease associations of common DEGs between males and females. Canonical pathway analysis was conducted using GeneTrial2 online database [38,39]. The heat maps of DEGs were constructed with PermutMatrix software [40,41].

The significant associations (corrected *p* values < 0.05) between identified differentially expressed genes with human diseases were analyzed using the Comparative Toxicogenomics database [42]. This database contains literature-based, manually curated interactions that are integrated to create a knowledge base that interrelates chemical, gene, phenotype, disease, and exposure information.

### 2.9. Cognitive Function Assessment

At the end of dietary feeding, 32-week-old male and female mice from each of the 4 diet/inhibitor treatment groups (LGD, HGD, LGD + sEHI, and HGD + sEHI), *n* = 7 mice/group, were subjected to a cognitive function test using Y-maze spontaneous alternation behavior (SAB) assessment. Y-maze is a behavioral test for measuring the willingness of rodents to explore new environments and a commonly used tool to measure of spatial working and short term memory in mice. Mice were adapted to the testing room for 30 min, then placed in the center of the Y-shaped maze with three white plastic arms (35L × 8W × 15H cm) at 120° angles from each other. Mice were allowed to freely explore the three arms of the Y-maze for 8 min and tracked with an overhead camera to record the number of entries into each arm. Entry into each arm (arm 1–3) after entry to the center (arm 4), total distance traveled, latency to entry and frequency, and an alternation score was computed as the number of times the three arms were sequentially entered. Data were expressed as the % alternation score, the number of alternations divided by maximum alteration triplets and presented as means ± SEM. The Y-maze studies were performed by the UC Davis Mouse Biology Program, Phenotyping Center (MMPC).

## 3. Results

### 3.1. Sex Differences in Weight, Glucose and Insulin

All of the study mice consumed similar amounts of the various diets. Water consumption was measured and there were no differences in the amount of water consumed by mice in the experimental diet/inhibitor groups. As expected, WT male mice weighed more than WT females across all baseline and post-diet comparisons in the presence or absence of the sEHI (*p* < 0.05), Supplemental Table S2.

Fasting serum glucose levels were higher in males than females (*p* < 0.05) on the LGD, while there were no sex differences in glucose levels for mice on the HGD. For mice receiving the sEHI, there were no sex differences in fasting glucose levels in either diet group. Fasting serum insulin levels did not differ between males and females regardless of diet or inhibitor treatment (Supplemental Table S3).

There was no difference in estrous cycle phase for female mice in the dietary or inhibitor study groups.

### 3.2. Comparison of Global Gene Expression Profiles between Male and Female Mice

**Figure 1:**
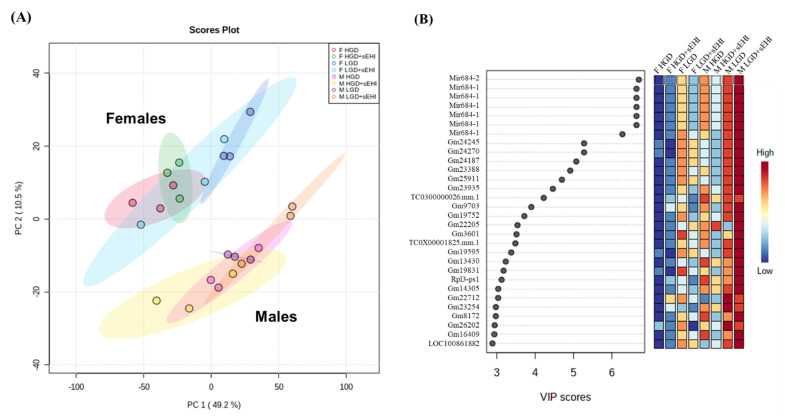
Principal component analysis (PCA) and variable importance in projection (VIP) scores of hippocampal microvascular gene expression in female and male mice. (**A**) The PCA plot illustrates the trends of the global gene expression in the hippocampal microvessels of the females (F) and males (M) in the following diet/inhibitor groups: high glycemic diet (F HGD, red and M HGD, pink), HGD with soluble epoxide hydrolase inhibitor (F HGD + sEHI, green and M HGD + sEHI, yellow), low glycemic diet (F LGD, dark blue and M LGD, purple), F LGD + sEHI (light blue), and M LGD + sEHI (orange). Most significant differences in the dataset are displayed as percent of the total variance using principal component 1 (PC1) and PC2 on the x- and y-axes, respectively. Three biological replicates per each female or male diet/inhibitor group. (**B**) VIP Scores for the top 30 genes responsible for the separation between females and males in the diet/inhibitor groups obtained using PLS-DA. The x-axis represents the VIP scores for each gene (black circles) shown on the y-axis. Color box gradation from blue to red indicates low to high levels of gene expression, respectively.

Principal component analysis (PCA) allows one to visualize relatedness between global genomic profiles of the studied populations, the results of which are usually discussed in terms of component scores for each population studied. The PCA analyses showed that the expressed profiles of genes of our experimental diets and inhibitor groups were distinctly different in females compared to males, Figure 1A. The genes with the highest variables importance in projection (VIP) scores that contributed to the separation between male and female genomic profiles revealed by partial least squares-discriminant analysis (PLS-DA) analysis, Figure 1B.

**Figure 2:**
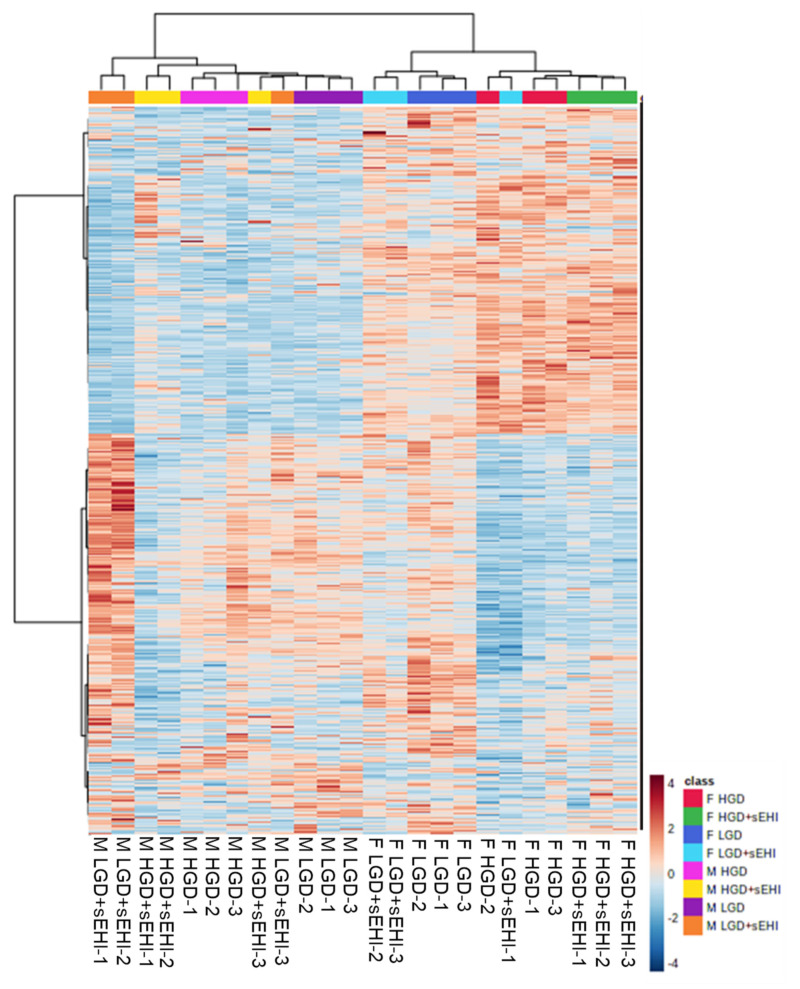
Hierarchical clustering of global gene expression profiles in female and male mice. Heat map of the signal intensity of genes expressed in the hippocampal microvessels of female (F) and male (M) mice. The rows of the heat map indicate the individual signal intensities of genes, while the color boxes at the top of the columns represent the following sex (F female, M male), diet groups (HGD high glycemic diet, LGD low glycemic diet), and with or without a soluble epoxide hydrolase inhibitor (sEHI): high glycemic diet (F HGD, red and M HGD, pink), F HGD + sEHI (green), M HGD + sEHI (yellow), low glycemic diet (F LGD, dark blue and M LGD, purple), F LGD + sEHI (light blue), and M LGD + sEHI (orange). Three biological replicates per each female or male diet/inhibitor group. Genes with lower signal intensities are shown in blue, and genes with higher signal intensities are shown in orange on the heat map.

Among the top 30 genes with the highest VIP scores were mir-684-1, mir-684-2, Gm24245, Gm24270, Gm24187, Gm23388, Gm25911, Gm23935, and Rpl3 or Gm23254. These VIP scores were from genes belonging to different categories, including microRNAs (miRNAs), protein coding RNAs, or small nucleolar RNAs (snoRNAs), suggesting that these different RNA types play an important role in differences in the response to the glycemic diet between male and female mice. Together with PCA, the two-dimensional hierarchical clustering of global gene expression profiles. Figure 2 also showed differences in profiles between male and female mice. This suggests that the effect of the hyperglycemic diet and that of the soluble epoxide hydrolase inhibitor is fundamentally different at the genome level in male and female mice.

### 3.3. Sex Differences in Differentially Expressed Protein Coding and Non-Coding RNAs

**Figure 3:**
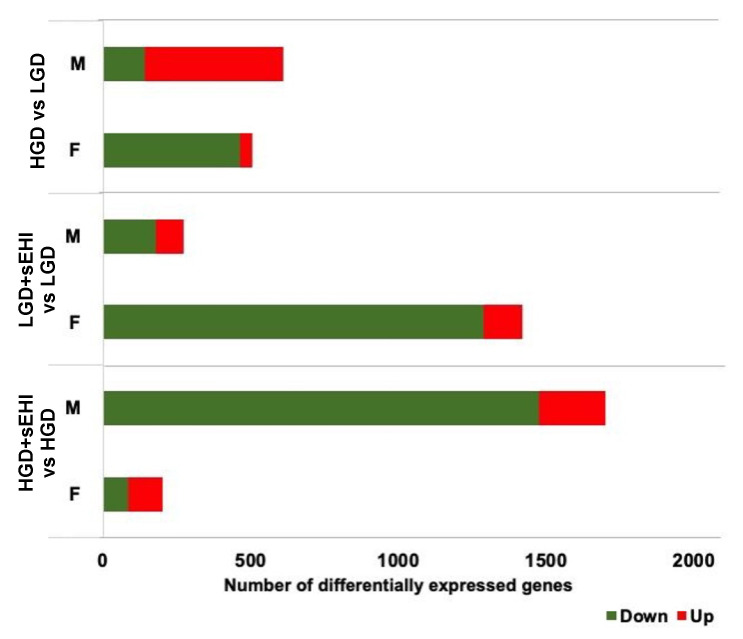
Sex differences in differentially expressed genes (DEGs) in the hippocampal microvessels for the low glycemic diet (LGD) and the high glycemic diet (HGD) with and without soluble epoxide hydrolase inhibitor (sEHI). Histogram shows the number of upregulated (red) and downregulated (green) differentially expressed genes (DEGs) between females (F) and males (M) in the following three diet/inhibitor comparisons: high glycemic diet (HGD) compared to low glycemic diet (LGD), LGD with soluble epoxide hydrolase inhibitor (LGD + sEHI) compared to LGD alone, and HGD + sEHI compared to HGD alone.

We also observed that biologic sex exerted an effect on differential RNA expression depending on diet. When mice on the HGD were compared to those on the LGD, the total number of differentially expressed genes (DEGs) was similar in males and females. However, the majority of DEGs were upregulated by the HGD in males, while the HGD resulted in mostly downregulated DEGs in females. For mice consuming the LGD, the sEHI had a greater effect in females than in males with 1420 DEGs identified in females compared to only 272 DEGs in males (Figure 3). In contrast, in mice consuming the HGD, the sEHI had a more pronounced effect on the transcriptome of males (1701 DEGs) than that of females (200 DEGs) (Figure 3).

**Figure 4:**
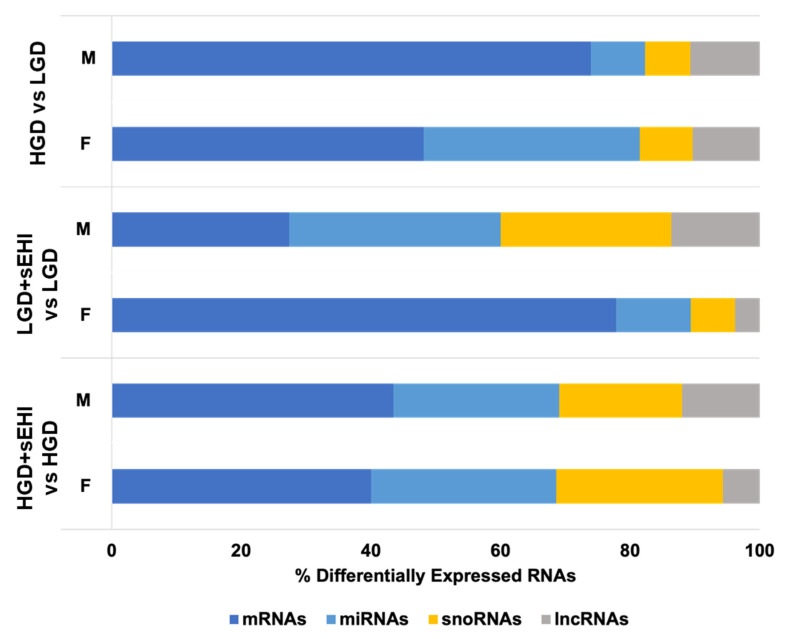
Sex differences in the percentage of differentially expressed protein coding and noncoding RNAs in the hippocampal microvascular endothelium of female and male mice. Histogram illustrates the percentage of differentially expressed RNAs in the hippocampal microvasculature of female (F) and male (M) mice in the following three diet/inhibitor comparisons: high glycemic diet (HGD) compared to low glycemic diet (LGD), LGD with soluble epoxide hydrolase inhibitor (LGD + sEHI) compared to LGD alone, and HGD + sEHI compared to HGD alone. Protein coding messenger RNAs (mRNAs, dark blue), and noncoding RNAs including microRNAs (miRNAs, light blue), small nucleolar RNAs (snoRNAs, yellow), and long noncoding RNAs (lncRNAs, grey).

The differences in genomic changes were not only observed in the number of genes presenting changes in their expression but also in the types of RNAs. Nearly 74% of DEGs were identified as protein coding in the HGD vs. LGD comparison for males, whereas 48% of DEGs were protein coding for the same comparison in females. In addition, among the noncoding RNAs, DEGs for mice on the HGD were composed of a greater number of miRNAs in females (33%) than in males (8%). The biggest difference was observed in response to the sEH inhibitor on the LGD where DEGs made up of 27% protein coding genes, 33% miRNAs, 26% snoRNAs, and 14% long noncoding RNAs (lncRNAs) in males, while resulting in DEGs composed primarily of protein coding mRNAs (78%), and a lesser percentage of non-coding RNAs (12% miRNAs, 7% snoRNAs, and 3% lncRNAs) in females (Figure 4). Thus, both the protein coding RNAs and noncoding RNAs were modulated by the high and low glycemic diets, with or without the sEH inhibitor, in a sex-dependent manner.

### 3.4. Functional Analysis of the Differentially Expressed Genes in Female and Male Mice

**Figure 5:**
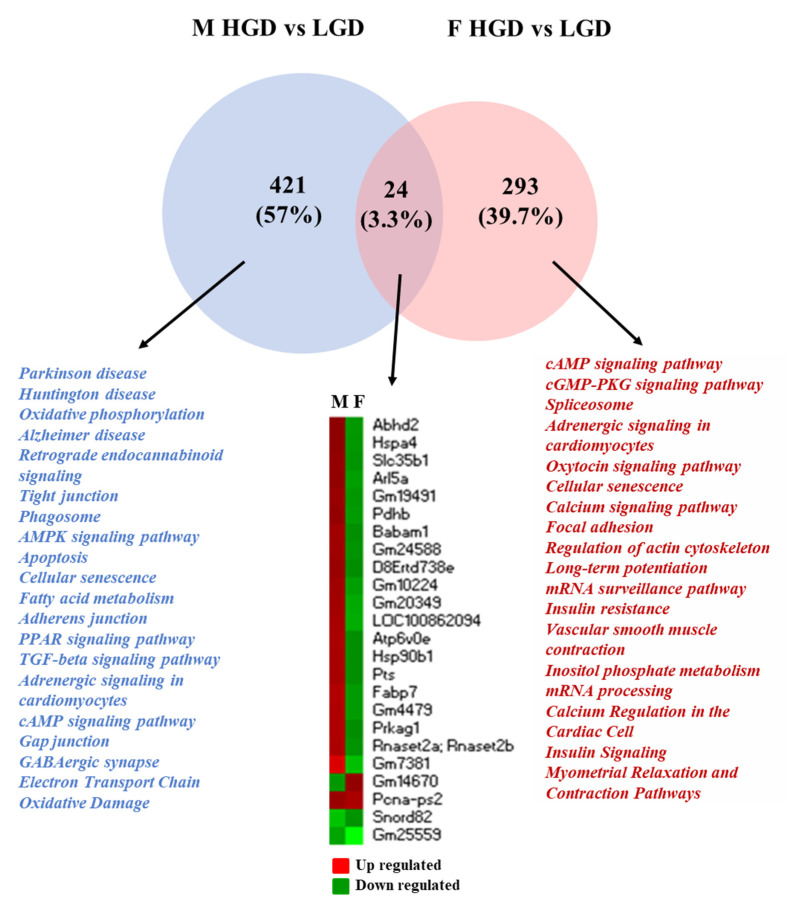
Sex differences in cellular pathways modulated by the differentially expressed genes in the hippocampal microvessels for the high glycemic diet (HGD) compared to the low glycemic diet (LGD). Venn diagram shows the cellular pathways modulated by the differentially expressed genes (DEGs) specific to males (*n* = 421, blue) and specific to females (*n* = 293, pink) in the hippocampal microvessels from high glycemic diet (HGD) compared to the low glycemic diet (LGD)-fed wild type mice. The few (*n* = 24) DEGs in common (dark pink in the Venn diagram) between males and females are shown in the heat map (upregulated genes are shown in red, downregulated genes are shown in green).

The next step of our analysis was to compare DEGs and their cellular functions in males and females. When comparing the HGD to the LGD (Figure 5), we identified 24 genes in common between males and females, with 421 DEGs specific to male mice (mostly upregulated, Figure 3) and 293 DEGs specific to female mice (mostly downregulated, Figure 3).

The majority of the common genes were upregulated in males while they were downregulated in females. Using the Enrichr tool, we identified that these common DEGs with opposite expression profiles were associated with diseases such as coronary artery disease, blood pressure, and anxiety disorders (Supplemental Table S4). The pathway analysis of male and female specific genes revealed that they were involved in the regulation of different cellular functions. The male specific genes modulated by the HGD were involved in pathways regulating neurodegenerative diseases, metabolism, electron transport chain, or cell signaling. On the other hand, genes specific to females were involved in the regulation of endothelial functions, such as focal adhesion and actin cytoskeleton or cell signaling, including calcium signaling and insulin resistance. Both male- and female-specific DEGs were involved in cellular senescence pathways. Thus, under hyperglycemic stress in the hippocampal microvasculature, the upregulated genes unique to males were mostly involved in neurodegenerative diseases, while the downregulated genes unique to females were involved in pathways primarily related to endothelial function.

**Figure 6:**
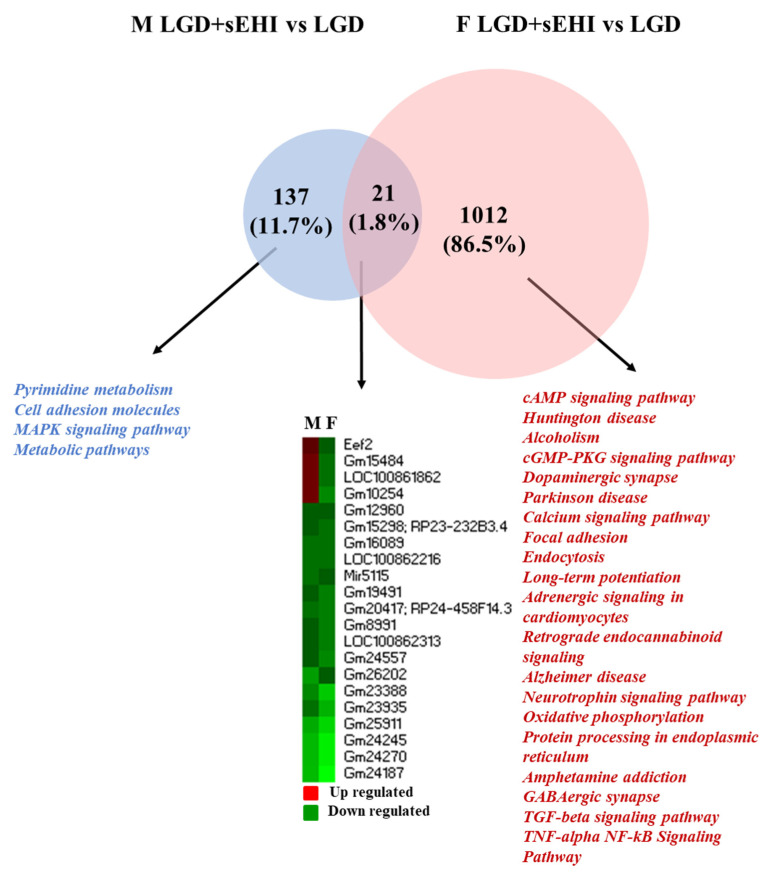
Sex differences in cellular pathways modulated by the differentially expressed genes in the hippocampal microvessels for the low glycemic diet (LGD) with and without the soluble epoxide hydrolase inhibitor. Venn diagram shows the cellular pathways modulated by the differentially expressed genes (DEGs) specific to males (*n* = 137, blue) and specific to females (*n* = 1012, pink) in the hippocampal microvessels from low glycemic diet (LGD) with soluble epoxide hydrolase inhibitor (sEHI)-fed mice compared to the LGD alone. The few (*n* = 21) DEGs in common (dark pink in the Venn diagram) between males and females are shown in the heat map (upregulated genes are shown in red, downregulated genes are shown in green).

The comparison of genes identified as modulated by the sEHI on the LGD identified 21 genes in common between males and females (Figure 6). Their expression was mostly downregulation in both the sexes (Figure 3). These common DEGs were associated with diseases including Alzheimer’s disease and cerebellar degeneration (Supplemental Table S4).

**Figure 7:**
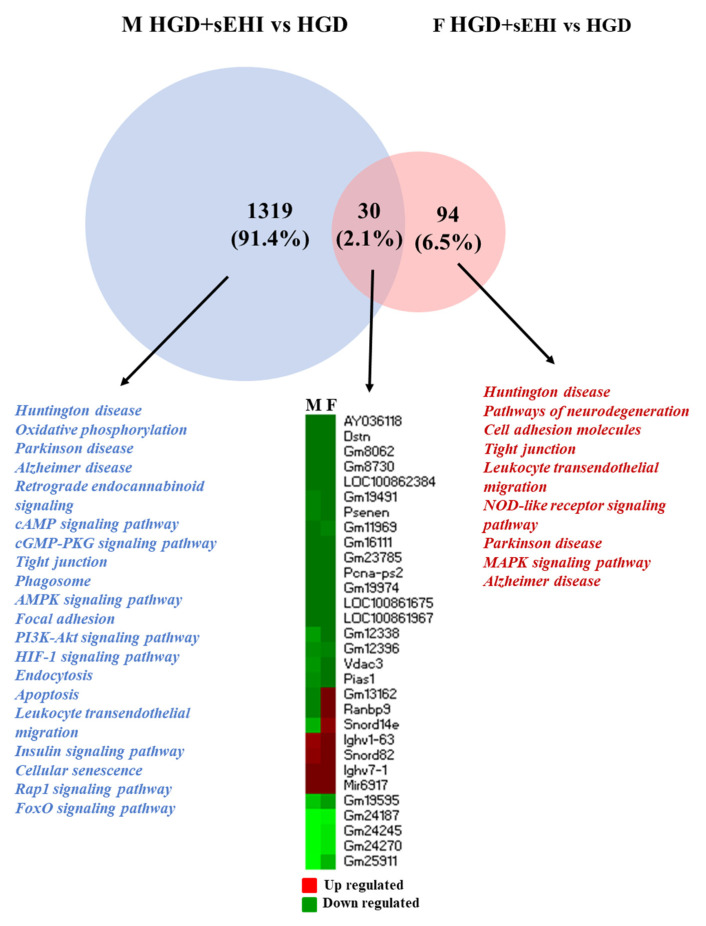
Sex differences in cellular pathways modulated by the differentially expressed genes in the hippocampal microvessels for the high glycemic diet (HGD) with and without the soluble epoxide hydrolase inhibitor. Venn diagram shows the cellular pathways modulated by the differentially expressed genes (DEGs) specific to males (*n* = 1319, blue) and specific to females (*n* = 94, pink) in the hippocampal microvessels from mice fed the high glycemic diet (HGD) with soluble epoxide hydrolase inhibitor (sEHI) compared to without sEHI. The few (*n* = 30) DEGs in common (dark pink in the Venn diagram) between males and females are shown in the heat map (upregulated genes are shown in red, downregulated genes are shown in green).

However, fewer pathways were identified for male specific genes, although the number of genes which had a significant change in expression was relatively small. On the other hand, a large number of pathways were identified for female specific genes. These pathways were involved in the regulation of cell signaling, neurodegenerative diseases, and endothelial function. Thus, with the LGD, the impact of the inhibitor on brain microvascular function was greater in female mice compared to males. In contrast, with the HGD, the inhibitor affected the expression of a large number of genes in males and very few genes in females, with 30 genes modulated in common (Figure 7). The interrogation of disease databases with the Enrichr software revealed that these DEGs in common between males and females were involved in memory related disorders, blood pressure, and coronary artery disease (Supplemental Table S4).

### 3.5. Sex Differences in Transcription Factors

**Figure 8:**
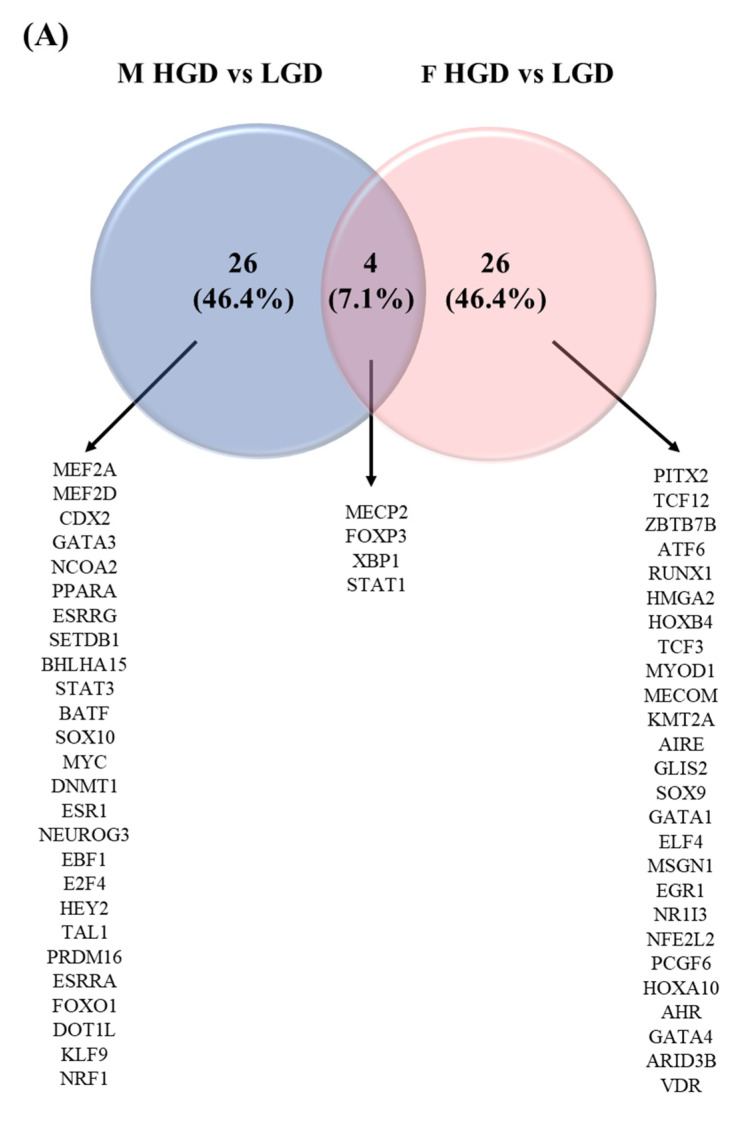

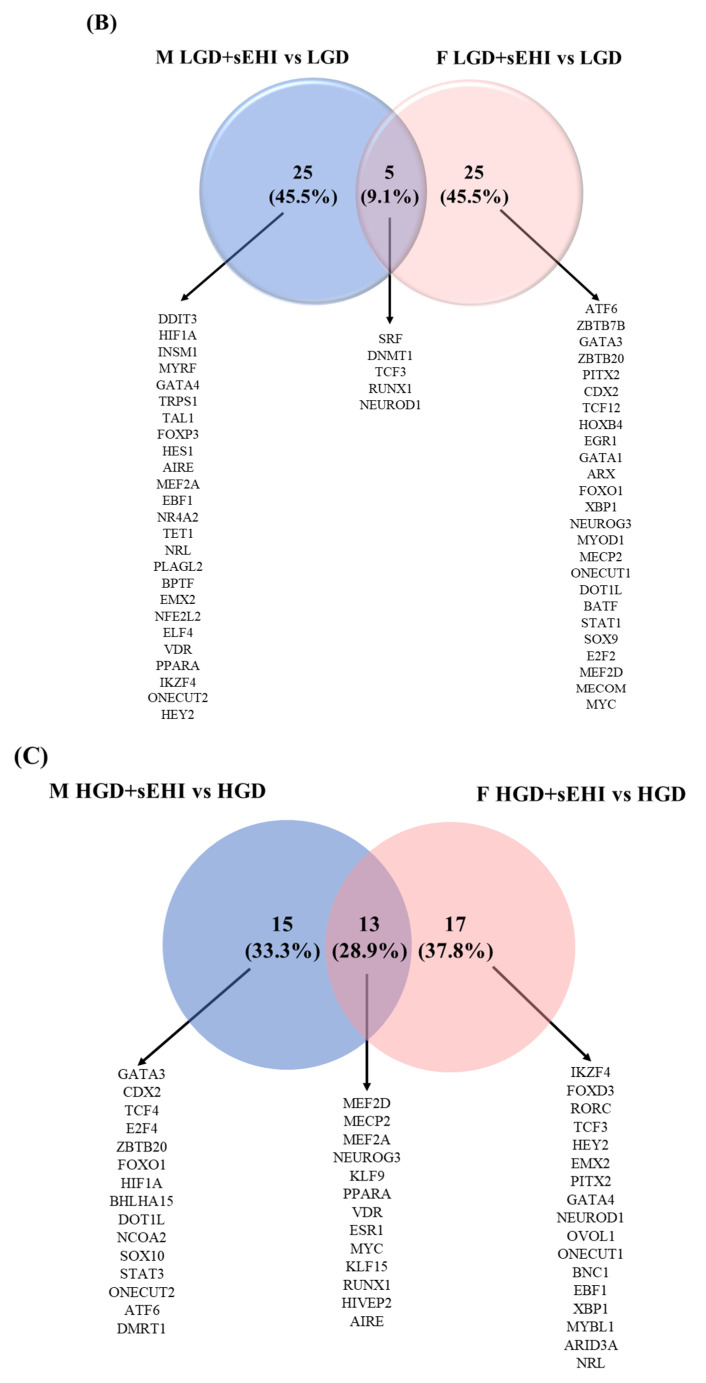
Venn diagrams of the top 30 transcription factors affected by diet and inhibitor in the hippocampal microvasculature of female and male mice. (**A**) Venn diagram comparing the transcription factors (TFs) in the hippocampal microvessels of female (F) and male (M) mice for the high glycemic diet (HGD) compared to the low glycemic diet (LGD) shows 26 female specific TFs (pink), 26 male specific TFs (blue), and 4 TFs in common (purple). (**B**) Venn diagram comparing the TFs in the hippocampal microvessels of female (F) and male (M) mice for the LGD with soluble epoxide hydrolase inhibitor (sEHI) compared to without sEHI shows 25 female specific TFs (pink), 25 male specific TFs (blue), and 5 TFs in common (dark pink). (**C**) Venn diagram comparing the TFs in the hippocampal microvessels of female (F) and male (M) mice for the HGD + sEHI versus HGD comparison shows 17 female specific TFs (pink), 15 male specific TFs (blue), and 13 TFs in common (dark pink).

In order to explain differences in the observed genomic effects in response to the HGD and the sEHI, we identified and compared potential transcription factors involved in the observed genomic modifications. Venn diagrams were constructed for transcription factors (TFs) for females and males for all the diets and inhibitor groups. We observed that there were four common TFs between males and females on the HGD compared to the LGD, including STAT1 and FOXP3 (Figure 8A). These TFs were involved in neuroinflammation, apoptosis, and neurodegeneration. However, the majority of TFs were different and specific between males and females. When restricting the analysis to the top thirty TFs in males and females for the inhibitor on the LGD, five TFs were identified in common, including DNMT1, and NEUROD1 or RUNX1 (Figure 8B). These TFs were associated with the epigenetic modifications of vascular endothelium, hippocampal neurogenesis, and endothelial cell adhesion. The remaining TFs, however, were either specific to males or unique to females. On the other hand, in mice on the HGD with the inhibitor, 13 TFs were in common between males and females, including KLF15, MEF2A, or PPARA, Figure 8C. They were involved in the regulation of vascular inflammation, neuronal survival, and cell metabolism. Interestingly, RUNX1 was found in common between males and females with the inhibitor for both the LGD and the HGD. Thus, sex differences in the identified transcription factors following the chronic consumption of a HGD could explain the differences in the observed genomic effects between males and females.

### 3.6. Disease Associations of Differentially Expressed Genes in Female and Male Mice

**Figure 9:**
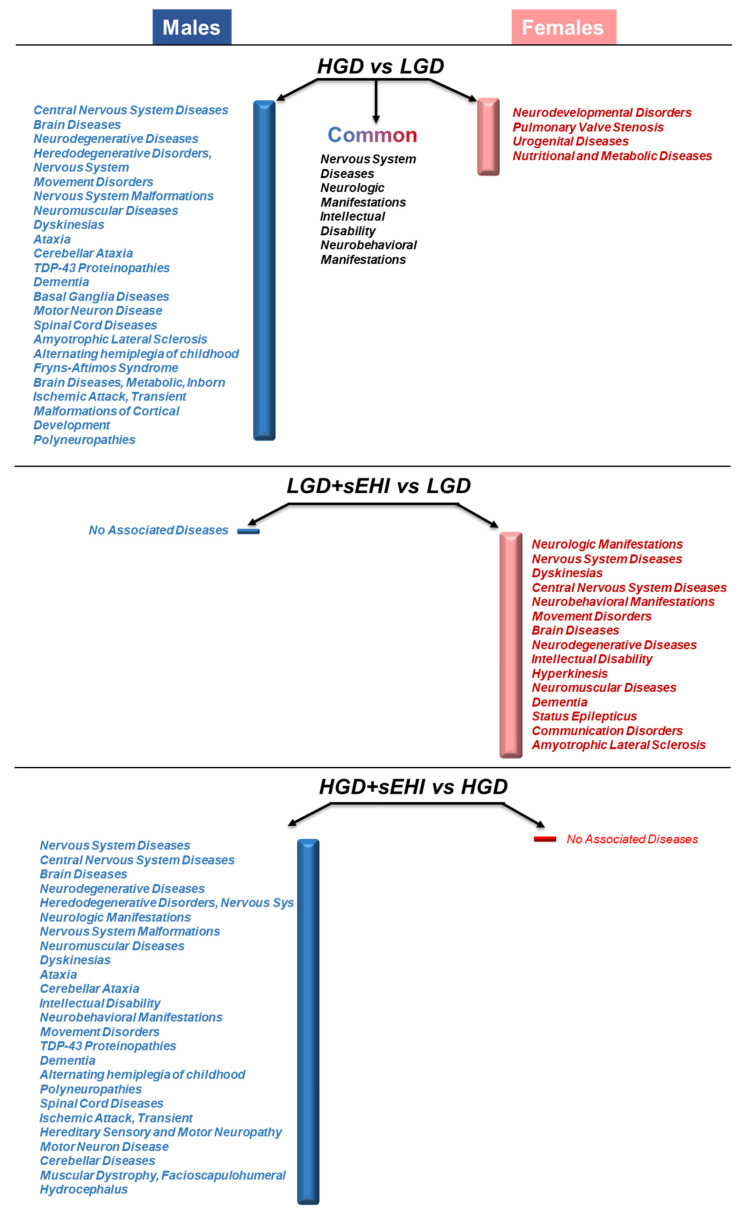
Sex differences in the disease associations of differentially expressed genes in the hippocampal microvascular endothelium of female and male mice. Diseases associated with genomic modifications in the hippocampal microvasculature of females and males are presented next to blue and pink bars for males and females, respectively, for the following diet/inhibitor comparisons: the high glycemic diet (HGD) versus low glycemic diet (LGD), the LGD with soluble epoxide hydrolase inhibitor (sEHI) compared to the LGD alone, and the HGD + sEHI versus the HGD alone. Associated diseases were identified using Toxicogenomics database—a novel platform that connects toxicological data for genes, phenotypes, diseases, chemicals, and other exposures by integrating manually curated interactions with literature-based interactions and providing a synchronized heterogenous information.

Since our studies were conducted only in mice, our next step was to identify clinical diseases associated with the differentially expressed genes in the diet/inhibitor comparison groups in males and females by interrogating the Toxicogenomic database, a premier public resource for literature-based, manually curated data, which assesses the effect of environmental stressors on human health at the genetic level. This analysis showed that the genes detected as differentially expressed in the HGD vs. LGD comparison in males were associated with over 20 clinical diseases, including central nervous system diseases, brain diseases, or ischemia. In contrast DEGs in females on the HGD compared to the LGD, were associated with eight diseases, including neurodevelopmental disorders and nutritional and metabolic diseases (Figure 9). The comparison of diseases showed that there were four in common between males and females, namely, nervous system diseases, neurologic manifestations, intellectual disability, and neurobehavioral manifestations. This observation suggests that the HGD had a higher impact on molecular targets associated with clinical diseases in males than in females and on several neurodegenerative diseases commonly observed in both males and females.

With regards to the inhibitor, in females the sEHI-induced DEGs for the LGD were potentially associated with 15 diseases, including neurodegenerative diseases or dementia. On the other hand, in males, no associated diseases were found for the LGD + sEHI when compared to the LGD alone (Figure 9). However, this difference could be explained by the fact that the number of DEGs was greater in females compared to males on the LGD with sEH inhibitor compared to the LGD alone (Figure 3). In contrast, genomic modifications for the HGD with inhibitor compared to without sEHI in male mice were related to 25 diseases, including brain diseases, neurological manifestations, or cerebellar ataxia, while female mice on the HGD with the sEHI showed very little effect on differential gene expression with no associated diseases found (Figure 3).

### 3.7. Sex Differences in Cognitive Function

We used Y-maze to evaluate the cognitive performance in male and female mice. The Y-maze determines spatial cognition (hippocampal), learning, and memory in mice by calculating alternating triplets (animal visits to all three arms of the Y-maze in sequence). Cognition was assessed for differences in spontaneous alternation behavior (SAB) in the Y-maze connected to the number of arm entries by mean % alternation triplets (# alternating triplets/total # triplets). We did not identify any statistically significant differences in cognitive function as assessed by Y-maze for the HGD-fed males (45.8 ± 0.7% alternation triplets) and females (48.7 ± 2.2% alternation triplets) when compared to the LGD-fed male (44.0 ± 1.5% alternation triplets) and female mice (44.3 ± 2.4% alternation triplets), respectively. We observed statistically improved memory performance (*p* < 0.05) following the LGD with the soluble epoxide hydrolase inhibitor (sEHI) in males (58.0 ± 1.9% alternation triplets) and females (58.0 ± 4.2% alternation triplets) when compared to the LGD alone. Compared to the HGD alone, we observed statistically improved memory performance (*p* < 0.05) in males following the HGD with the sEHI (56.4 ± 0.6% alternation triplets). However, there were no statistically significant differences in the cognitive function of female mice on the HGD + sEHI (57.5 ± 5.5% alternation triplets) compared to the HGD. Thus, cognitive performance measured by Y-maze improved with the sEH inhibitor on the LGD condition in both males and females. In contrast, the sEH inhibitor on the HGD enhanced the cognition in only males.

## 4. Discussion

In this study, we aimed to characterize the multi-omic and sex-based response of differential gene expression in the brain microvasculature of the wild type murine hippocampus, an important memory center, following chronic exposure to a high glycemic diet with and without a soluble epoxide hydrolase inhibitor. The strength of our study is the use of non-targeted multi-genomic analysis, which allowed us to access the expression of the entire mouse genome. Use of such a state-of-the-art approach also permitted the comprehensive identification of novel targets underlying responses to HGD conditions and how they were modified by the inhibition of sEH. Indeed, our genomic analyzes identified multiple previously undescribed genes whose expression was differentially affected by a HGD and revealed how the response was modified by a soluble epoxide hydrolase inhibitor (sEHI). Our work also revealed and summarizes striking sex specific differences between males and females.

Differences in the genomic response to diets and nutrients depending on sex/gender have been reported in a few prior studies. The sex/gender-specific genomic studies are crucial to generate the data required for the precision nutrition development that will make more personalized dietary recommendations to improve health and prevent disease progression in the future [43]. We have previously shown that lipotoxicity induces different genomic modifications between male and female mice in brain microvasculature [9]. Similarly, a sexually dimorphic transcriptome has been reported in pancreatic β cells following a high-fat diet-induced type-2 diabetes mouse model [44]. Sexual dimorphism in hepatic gene expression in response to dietary carbohydrate has also been observed in zebrafish [45]. Furthermore, gender-dependent changes in the expression of genes has also been described for other nutrients, such as micronutrients. For example, in peripheral blood mononuclear cells, a significant difference in response to curcumin intake has also been reported between male and female volunteers [46].

Below we discuss our findings in the context provided above and in terms of examples of some of the important differentially expressed genes by the HGD in males and females, genes in common, transcription factors that may explain the sex differences, and clinical correlates to human disease.

### 4.1. Differentially Expressed Genes by the HGD in Males and Females

Among the male-specific differentially expressed genes upregulated by the HGD, was IGFBP7 (Insulin-Like Growth Factor Binding Protein 7). The increased expression of IGFBP7 is observed in the vasculature of different brain pathological conditions, such as stroke [47], traumatic brain injury [48], or multiple sclerosis [49], suggesting that IGFBP7 may be a general marker of the vascular response to pathological conditions in the brain [48,50]. Another male specific DEG upregulated by the HGD was UQCR11 (Ubiquinol-Cytochrome C Reductase, Complex III Subunit XI). UQCR11 plays an important role in the conversion of mild cognitive impairment to Alzheimer’s disease [51] and is associated with mitochondrial dysfunction [52]. Among the female specific DEGs downregulated by the HGD was NOSTRIN (Nitric Oxide Synthase Trafficking). The elevated levels of NOSTRIN are linked to white matter hyperintensity in aging brains [53]. Another DEG whose expression was decreased in female mice fed the HGD was CAMK4 (Calcium/Calmodulin Dependent Protein Kinase IV). CAMK4 is upregulated in Alzheimer’s disease patients [54] and associated with mitochondrial dysfunction and neuroinflammation. In addition, the increased expression of CAMK4 is involved in neurodegeneration in rat hippocampal cells [55]. Taken together, the HGD upregulated male specific DEGs may contribute to mitochondrial dysfunction and neurodegeneration in the hippocampal microvasculature. In contrast, the female specific DEGs downregulated by the HGD appear to be protective against neuroinflammation, mitochondrial damage, and neurodegeneration.

### 4.2. Genes in Common between Males and Females

Our bioinformatics analysis identified genes differentially expressed in both males and females in each of the diet and inhibitor comparison groups, suggesting that the regulation of these genes is important to each condition. Among the common differentially expressed genes in the HGD vs. LGD dietary comparison group, though with opposite expression patterns (upregulated in males and downregulated in females), was ARL5A (ADP Ribosylation Factor-Like GTPase 5A). This gene is differentially expressed in the brains of early and late Alzheimer’s disease [56]. Another gene in common was ABHD2 (Abhydrolase Domain Containing 2, Acylglycerol Lipase), which is known to interact with proteins associated with neurodegeneration, type 2 diabetes, and stroke [57]. Among the common differentially expressed genes in the LGD with sEHI was EEF2 (Eukaryotic elongation factor 2), though upregulated in males but downregulated in females. EEF2 is a key regulator of protein synthesis and its suppression in the hippocampus has been shown to improve contextual fear memory in mice [58,59]. In the HGD with sEHI, the gene expression of RanBP9 (Ran-binding protein 9) was also in common in males and females but had opposite expression profiles (downregulated in males, whereas upregulated in females). The overexpression of RanBP9 is associated with diminished learning and memory in Alzheimer’s disease mouse models [60]. Thus, the common genes modulated by the HGD in males and females had opposite expression patterns for genes that may promote neuroprotection in females and neurodegeneration in males.

### 4.3. Transcription Factors That May Explain the Sex Differences

Our bioinformatic analyses of novel genomic data allowed us not only to identify genes with changes in expression depending on diet and/or sex, but also potential transcription factors, which are likely regulate the expression of the differentially expressed genes identified. Among the transcription factors identified in our study was RUNX1, which probably functions as a key central regulatory gene in the brain hippocampal microvasculature since it was differentially regulated in both the LGD and HGD diets and in both sexes. The RUNX1 gene codes for a namesake protein, runt-related transcription factor 1 (RUNX1). RUNX1 binds to the core element of many enhancers and promoters and can accelerate apoptosis [61]. It has also been postulated to represent a paradigm for transcriptional and epigenetic reprogramming through a multi-layered interaction between RUNX1, epigenetic modifiers, and the epigenome [62]. Although this process has been described for RUNX1 in hematological malignancies [63], to our knowledge, ours is the first description of a key role for the RUNX1 transcription factor in response to chronic high glycemic dietary stress. An additional differentially expressed TF identified in females in our study was SOX9 (SRY-Box Transcription Factor 9), which has been reported to have a potential protective role against cerebral ischemia/reperfusion injury [64] and has been shown to present a sex-dependent mode of expression in different tissues [65]. An additional transcription factor identified in our work and not previously reported to have sex-specificity was MEF2 (Myocyte Enhancer Factor 2), which was identified as being differentially expressed in males but not females in our study. MEF2 has been described as playing a key role in cognitive potential and conferring resilience to neurodegeneration [66]. Taken together, our findings are consistent with several transcription factors, including RUNX1, SOX9, and MEF2, as important transcription factors that may explain the sex differences in the brain microvascular response to high glycemic dietary stress. This is a new observation and an area in need of further study.

### 4.4. Clinical Correlates to Human Disease

The genomic data were used to identify potential diseases associated with the identified differentially expressed genes in our four experimental study groups (Figure 9). Several associated diseases were observed in common between male and female mice on the high glycemic diet (HGD), including nervous system diseases or neurobehavioral manifestations. However, a much greater number of diseases (22) were associated with DEGs in males compared to females. In males, diseases, such as neurodegenerative diseases, dementia, and cerebral ataxia, were associated with the DEGs, while in female mice, only four diseases were identified as associated with the differentially expressed genes and included neurodevelopmental disorders and metabolic diseases. This observation suggests that even though the number of genes modulated by the HGD was similar in both sexes (608 vs. 506 in males and females, respectively), the potential association with clinical disease was different between males and females and in general greater for males. This could be due to the fact that genes affected by the HGD in the microvasculature of the hippocampus are different, which indeed they are, suggesting that intake of a HGD can have more deleterious consequence on brain hippocampal microvasculature-associated diseases, such as dementia and neurodegeneration, in males than in females.

On the other hand, genes identified as modulated by the sEH inhibitor in females on the LGD were associated with a number of diseases, including neurologic manifestations, or intellectual disability, while no significant association with diseases were observed in males. This would suggest that the inhibition of soluble epoxide hydrolase in females, but not males, could be associated with clinical neurological diseases. This observation could be explained by the fact that the inhibitor affected a larger number of genes in females compared to males (1420 vs. 272). Interestingly, the opposite was observed for the inhibitor with the HGD, i.e., genes identified as modulated by the inhibitor in males on the HGD affected a large number of genes that were associated with numerous diseases, while the inhibitor modulated a much smaller number of genes in females on the HGD and no significant association with clinical disease was observed. This sex-dependent differences in response to a soluble epoxide hydrolase inhibitor could be due to sexually dimorphic regulation of epoxyeicosatrienoic acids (EETs) synthesis and metabolism by estrogen in response to endothelial dysfunction precipitated by an HGD. Specifically, since EETs are hydrolyzed by soluble epoxide hydrolase, decreases in sEH expression serve to potentiate EET bioavailability, responses that are reported to prevail in females as a function of estrogen [67].

### 4.5. Summary and Conclusion

Our sex-based analysis of male and female multi-omic expression data in response to high and low glycemic diets, with or without a soluble epoxide hydrolase inhibitor, led to the identification of previously unreported diet and sex differences in response to glycemic stress in brain microvasculature in mice. The HGD modulated the transcriptome of the male and female mice in opposite ways, by upregulating genes involved in mitochondrial dysfunction, neuroinflammation, and neurodegenerative diseases in males, while in females, the downregulation of genes was involved in these same pathways. This sexually dimorphic gene expression pattern may contribute to neuroprotection in females. In addition, the inhibition of soluble epoxide hydrolase had a primary effect on males on the HGD, mostly via the downregulation of DEG expression linked to functional pathways for neurodegeneration and apoptosis. In contrast, the effect of inhibition of soluble epoxide hydrolase was greater for females on the LGD, primarily via the downregulation of genes involved in pathways for cell signaling and endothelial function. Furthermore, a higher number of diseases were associated with the gene expression profiles of males on the HGD compared to females, suggesting that the HGD has a more deleterious effect on the male hippocampal microvasculature compared to females. Lastly, differential gene expression with the inhibitor on the HGD was linked to several neurodegenerative diseases, including dementia, in males, whereas no associations with clinical diseases were noted in females. Conversely, the DEGs modulated by the sEHI on the LGD were linked to neurological diseases in females but there were no disease associations in males.

The sex-specific response of neuroprotection in females under the conditions of hyperglycemic stress elucidated by our present work may provide explanations for some of the epidemiologic differences observed between males and females with Alzheimer’s disease (AD). As such, the sex-specific molecular mechanisms of regulation for the HGD and the sEH inhibitor identified in this study may provide future therapeutic molecular targets for the hyperglycemia-induced microvascular dysfunction associated with dementia, diabetes, and cardiovascular diseases.

## Data Availability

The microarray data in this study has been deposited in the GEO with the accession numbers GSE195975 and GSE185057.

## Supplementary Materials

The following supporting information can be downloaded at: https://www.mdpi.com/article/10.3390/nu14173451/s1, Table S1. Composition of the Low and High glycemic diets. Table S2. Mean body weight of wild type (WT) male and female mice on the high glycemic (HGD) and low glycemic (LGD) diets with or without soluble a soluble epoxide hydrolase inhibitor (sEHI). Table S3. Plasma Glucose and Insulins levels of wild type (WT) male and female mice post-diet intervention with the low glycemic diet (LGD) and high glycemic diet (HGD) with and without soluble epoxide hydrolase inhibitor (sEHI) treatment. Table S4. Sex differences in disease associations of differentially expressed genes in common between male and female diet/inhibitor comparison groups.
