# Supplementary material for: The Brain’s Microvascular Response to High Glycemia and to the Inhibition of Soluble Epoxide Hydrolase Is Sexually Dimorphic
Source: Nutrients. 2022 Aug 23;14(17):3451. doi: 10.3390/nu14173451 (PMC9460226; doi:10.3390/nu14173451)
Supplement: Supplementary file 1 [file nutrients-14-03451-s001.zip › nutrients-1798489-supplementary.pdf]

Table S1. Composition of the low and high glycemic diets.

**Component** (g/kg)	**LGD** (Teklad Envigo TD.08485)	**HGD** (Teklad Envigo TD.05230)
Casein	195	195
DL-Methionine	3	3
Sucrose	120	341
Corn Starch	432.99	211.99
Maltodextrin	100	100
Anhydrous Milkfat	37.2	37.2
Soybean Oil	12.8	12.8
Cellulose	50	50
Mineral Mix, (AIN-76 170915)	35	35
Calcium Carbonate	4	4
Vitamin Mix (Teklad 40060)	10	10
Ethoxyquin (antioxidant)	0.01	0.01

Table S2. Mean body weight of wild type (WT) male and female mice pre- and post-diet intervention with the high glycemic (HGD) and low glycemic (LGD) diets with or without soluble epoxide hydrolase inhibitor (sEHI).

Diet intervention	WT-LGD		WT-HGD		WT LGD+sEHI		WT HGD+sEHI	
	Male	Female	Male	Female	Male	Female	Male	Female
Baseline (Age 20 weeks)	30.5±1.5	23.8±1.0*	30.3±0.6	22.9±0.6*	31.5±1.1	27.4±1.1*	29.3±0.7	23.1±0.8*
Post Feeding (Age 32 weeks)	36.2±2.3	29.2±1.9*	34.3±0.9	26.2±1.3*	36.5±2.0	26.9±0.4*	34±1.1	24.3±0.5*

Data shown is mean ± SEM

* $p<0.05$ when compared to males in the same diet and inhibitor group.

Table S3. Plasma Glucose and Insulins levels of wild type (WT) male and female mice post-diet intervention with the low glycemic diet (LGD) and high glycemic diet (HGD) with and without soluble epoxide hydrolase inhibitor (sEHI) treatment.

	WT-LGD		WT-HGD		WT-LGD+sEHI		WT-HGD+sEHI	
	Female	Male	Female	Male	Female	Male	Female	Male
	Mean±SEM							
**Glucose (mg/dL)**	282.9±29.1*	416.1±41.0	311.0± 27.2	349.6± 36.8	319.4± 44.6	432.7± 48.6	448.6± 32.3	428±69.8
**Insulin (pg/dL)**	331.8±125.1	230.5±65.6	187.8± 27.9	215.4± 85.7	332.4± 64.4	285.4± 53.9	219.9±44.2	297.4± 70.9

* $p<0.05$ when compared to males in the same diet and inhibitor group

Table S4. Sex differences in disease associations of differentially expressed genes in common between male and female diet/inhibitor comparison groups.

**M HGD vs LGD compared to F HGD vs LGD**
Cognitive Therapy
Anxiety Disorders
Intelligence Tests
Arterial Pressure
Coronary Artery Disease
Blood Pressure
Response to cognitive-behavioral therapy in anxiety disorder
**M LGD+sEHI vs LGD compared to F LGD+sEHI vs LGD**
Progressive cerebellar ataxia
Cerebellar atrophy
Cerebellar degeneration
Alzheimer's Disease
**M HGD+sEHI vs HGD compared to F HGD+sEHI vs HGD**
Major depressive disorder
Coronary artery disease
Diastolic blood pressure
Memory
Migraine Disorders
